# Reference gene selection for gene expression study in shell gland and spleen of laying hens challenged with infectious bronchitis virus

**DOI:** 10.1038/s41598-017-14693-2

**Published:** 2017-10-27

**Authors:** Samiullah Khan, Juliet Roberts, Shu-Biao Wu

**Affiliations:** 1 Animal Science, School of Environmental and Rural Science, University of New England, Armidale, New South Wales 2351 Australia; 2 Present Address: School of Animal and Veterinary Sciences, The University of Adelaide, Roseworthy, South Australia 5371 Australia

## Abstract

Ten reference genes were investigated for normalisation of candidate target gene expression data in the shell gland and spleen of laying hens challenged with two strains of infectious bronchitis virus (IBV). Data were analysed with geNorm, NormFinder and BestKeeper, and a comprehensive ranking (geomean) was calculated. In the combined data set of IBV challenged shell gland samples, the comprehensive ranking showed TATA-box binding protein (*TBP*) and tyrosine 3-monooxygenase/tryptophan 5-monooxygenase activation protein zeta (*YWHAZ*) as the two most stable, and succinate dehydrogenase complex flavoprotein subunit A (*SDHA*) and albumin (*ALB*) as the two least stable reference genes. In the spleen, and in the combined data set of the shell gland and spleen, the two most stable and the two least stable reference genes were *TBP* and *YWHAZ*, and ribosomal protein L4 (*RPL4*) and *ALB*, respectively. Different ranking has been due to different algorithms. Validation studies showed that the use of the two most stable reference genes produced accurate and more robust gene expression data. The two most and least stable reference genes obtained in the study, were further used for candidate target gene expression data normalisation of the shell gland and spleen under an IBV infection model.

## Introduction

The five main segments of hen oviduct are ovary, infundibulum, magnum, isthmus and shell gland (uterus). The isthmus is involved mainly in shell membrane formation. The shell gland is involved in the synthesis and secretion of substances for the formation of distinct layers of the eggshell. During the egg formation cycle, an egg remains for approximately 18–20 hours in the shell gland during which shell formation takes place^1^. Calcium ions for shell formation are secreted from the shell gland cells and the *calbindin* gene plays a primary role in Ca^2+^ transportation^2^. Approximately 437 peptides and ion transporters have been identified as being involved in the formation of the eggshell^3,4^. Based on the role of the shell gland in synthesis of various components the eggshell, it is, metabolically, a very active organ in the reproductive tract of laying hens.

Infectious bronchitis virus (IBV) is a highly contagious mucosal pathogen of both broiler and layer chickens worldwide^5,6^. IBV replicates in cell cytoplasm and contains an un-segmented single stranded positive sense RNA of 27.6 kbp^7–9^. IBV has a short incubation period^6^, and viral spread occurs rapidly among chickens by aerosol and mechanical means^10,11^. IBV has the capability to multiply in various epithelial tissues, such as trachea^12,13^, kidney^14^, intestine^15,16^, spleen^17^ and oviduct^16,18–20^. The virus is well known for its effects in laying hens, including egg production and quality drops^10,16,21,22^. In Australia, there are two common forms of this virus, respiratory and nephropathogenic. Both types can induce various degrees of pathological changes in the oviduct of adult laying hens^20^. Genes involved in eggshell formation have been shown to be affected by IBV infection^23^. IBV infection induces a wide range of immune responses in chickens. An innate immune response is activated during the initial stages of infection in the mucosal lining of the trachea following binding of IBV virions to receptors on epithelial cells^24^. Activation of the innate immune response may be initiated by Toll-like receptors (TLRs) signalling upon IBV recognition^25^. Cellular and local immunity play a critical role in the protection of chicks from IBV infection^26^. Studies have shown that systemic immunisation generally fails to elicit strong mucosal immunity^27,28^. However, all ages are susceptible, with very young chicks exhibiting more severe respiratory signs and much higher mortality than older birds^29,30^. The spleen is a lymphoid organ that plays an important role in the initiation of the immune response against systemically induced antigens^31^ and is among the major organs where T and B cells are localized. In birds, the spleen serves as an important secondary immune organ as lymph nodes are not present^31,32^.

Quantifying gene expression in various patho-physiological conditions is a common technique in molecular biology. The two most commonly used methods of performing quantitative gene expression include relative and absolute quantification^33^. In relative quantification, qPCR data of candidate target genes of interest are achieved by including two or more most stably expressed internal control genes as an internal calibrator (reference genes)^34^. Selection of a reliable reference gene under the specific conditions is key to quantitative accuracy. The ideal reference gene should be expressed at a constant level in the tissue regardless of tissue nature, cell type, developmental stage and experimental conditions^34,35^. Traditionally, most commonly used housekeeping genes, such as *ACTB*, *TUBB* and *GAPDH* have been used widely as generic reference genes. However, ample evidence has shown that the expression of these genes may not be constant across a range of experimental conditions and tissues under investigation^36–38^. Thus, it is now recommended to use housekeeping genes as reference genes for normalisation only when prior analysis of their expression stability has been carried out. It is also recommended that more than one reference gene be used to achieve more robust, accurate and reliable normalisation of gene expression data^34^.

The programmes geNorm^34,39^, NormFinder^40,41^ and BestKeeper^42^ have been used to analyse the stability of housekeeping gene expression in samples from various sources. The underlying principle in each software is slightly different from the others and thus the resulting ranking of genes is not always the same. geNorm in qbase + module version 3.0 (Biogazelle, Belgium) calculates the gene expression stability (geNorm M) as the arithmetic mean of the pairwise variation (geNorm V) between all tested genes^34,39^. The geNorm V for any given two genes is the standard deviation calculated from the log_2_ transformed relative quantities between those two genes^34^ geNorm V shows level of variation in the average values of reference gene stability with the sequential inclusion of the next stable reference gene to the equation V_n/n+1_^34,39^. The analysis starts with the two most stably expressed genes being compared to the pair including the third (V2/3), and the process continues until the least stable gene is added (i.e. V9/10)^39^. To select the most stable single gene, geNorm re-calculates geNorm M after removing the least stable gene and repeats the process until the one most stable gene remains^39^. NormFinder calculates both inter- and intra-group variations and then combines the two to produce a stability value (SD), which thus represents a practical measure of the systemic error introduced when investigating the gene^40,41^. Hence, a low stability value reflects low inter- and intra-group variation^40,41^. An Excel based BestKeeper (Version 1.0) software determines the most stable reference genes based on Pearson correlation coefficient (r), coefficient of variance (CV) and standard deviation^42^. The current study investigated the expression stability of ten commonly used reference genes in laying hens infected with IBV T and Vic S strains. This study was performed in conjunction with a broader study in which the effect of IBV on the genes involved in eggshell formation in the shell gland and immune response in the spleen was investigated. The reference genes selected were then used for gene expression data normalisation of candidate target genes in the shell gland and spleen in an IBV model. Furthermore, different candidate target genes involved in calcium transportation (C*ALB1*) across cell membrane^43^ and protoporphyrin synthesis (*ABCB6*)^44^ in the shell gland and genes (*IFNγ* and *IL7*) involved in immune system in spleen of laying hens were used for the validation of the reference genes. The outcome of the study confirmed that *TBP* and *YWHAZ* were the two most stable reference genes in the shell gland and spleen tissues of chickens challenged with IBV.

## Results

### Efficiency and specificity of reference gene

**Figure 1:**
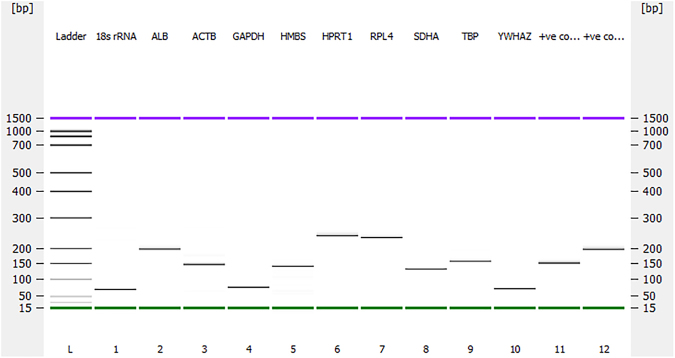
Amplification of the genes fragments from the shell gland tissue of chicken to assess the specificities of the primers used in the current study. (L) DNA ladder (bp); (**1**) *18 S rRNA* (63 bp); (**2**) *ALB* (197 bp); (**3**) *ACTB* (139 bp); (**4**) *GAPDH* (66 bp); (**5**) *HMBS* (131 bp); (**6**) *HPRT1* (245 bp); (**7**) *RPL4* (235 bp); (**8**) *SDHA* (126 bp); (**9**) *TBP* (147 bp); (**10**) *YWHAZ* (61 bp); (**11**) ND4-positive control (137 bp); (**12**) TLR7-positive control (200 bp). The upper (purple) and lower (green) markers act as internal standards and are used to align the ladder analysis with the individual DNA sample analysis. The standard curve (plotting migration time against DNA amplicon size), in conjunction with the markers, is then used to calculate DNA fragment sizes for each well from the migration times measured (see Agilent 2100 Bioanalyzer Users Guide for Molecular Assays). The DNA gel in Agilent 2100 Bioanalyzer was performed as per manufacturer’s instructions of Agilent DNA 1000 Kit.

**Figure 2:**
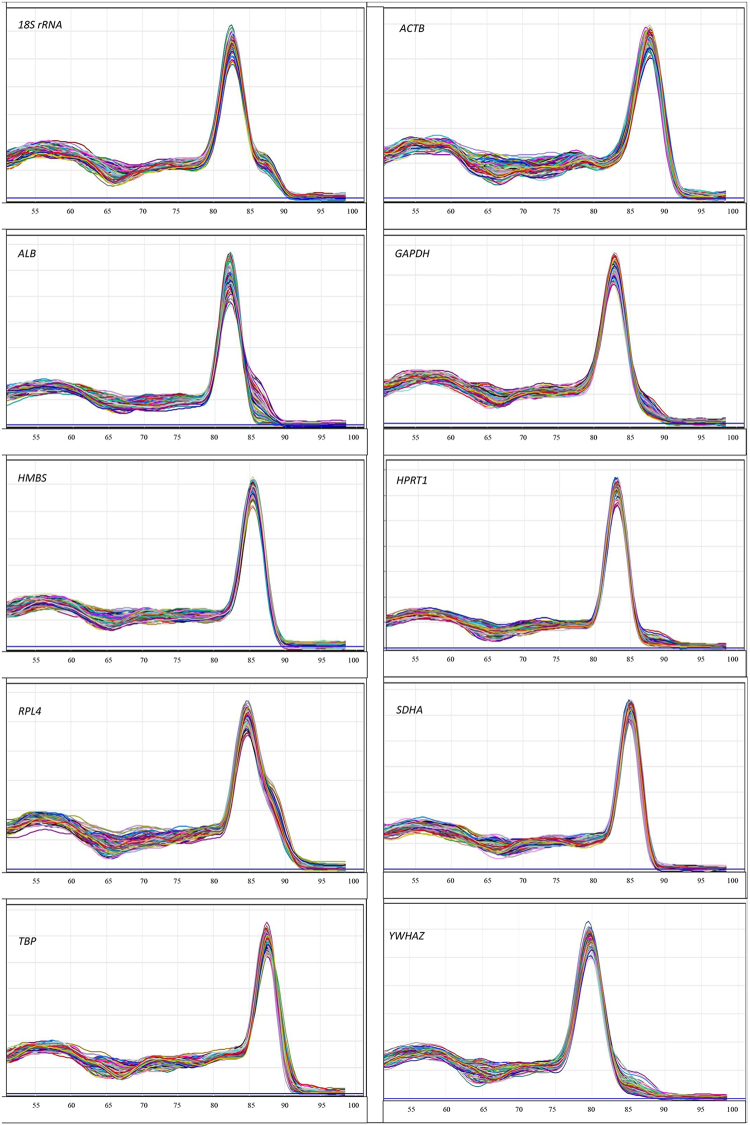
Melting curves of 10 reference genes in the shell gland samples. All melting curves showing a single peak indicated that all primers were specific in amplifying fragments of the genes and chances for primer dimers were minimum in the qPCR products. A melting phase at a ramp from 50 °C to 99 °C at 1 °C increments in Rotor-Gene Q was performed to assess the specificity of PCR amplification.

**Figure 3:**
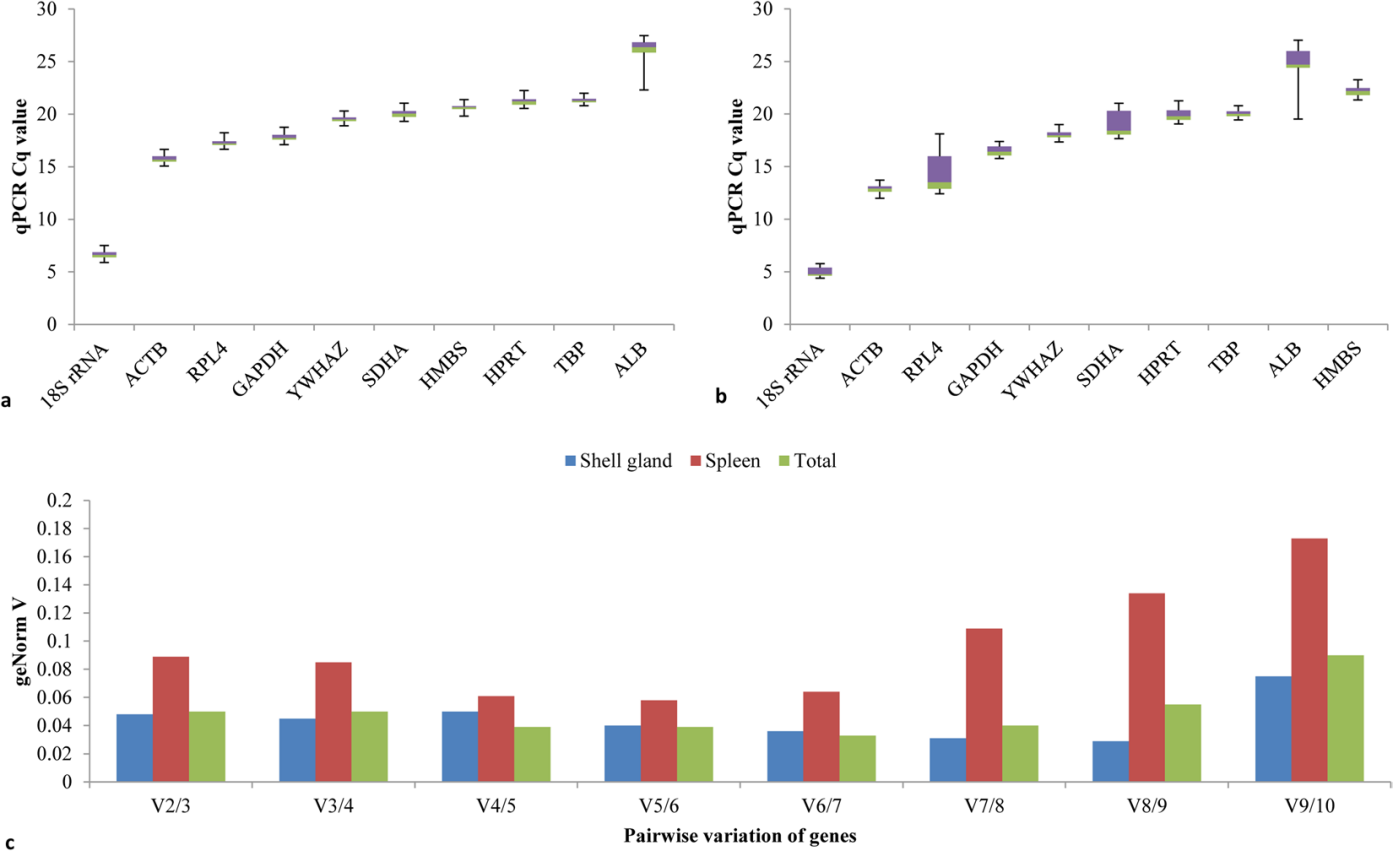
Effect of IBV challenge on the expression stability of 10 reference genes in the shell gland and spleen. Mean Cq values of the control, IBV T and Vic S strains challenged groups in the shell gland (**a**) and in the spleen (**b**). A square across the box is depicted as the median. The box indicates the 25th and 75th percentiles and the whiskers caps represent the maximum and minimum values (**c**) Pairwise variation (geNorm V) of genes in the shell gland (control, IBV T and Vic S strains challenge groups together), in the spleen (control, IBV T andVic S strains challenge groups together) and in the combined data set of the shell gland and spleen. In qbase + software, geNorm V was calculated as standard deviation of the log_2_ transformed relative quantities between those two genes. The geNorm V analysis started with the two most stably expressed genes being compared to the pair including the third (V2/3), and the process continued until the least stable gene was added (i.e. V9/10).

All primers amplified a single PCR product of the expected size confirmed by Agilent 2100 Bioanalyzer gel (Fig. 1). The melting curve analysis of all primer pairs further confirmed primer specificity and minimal primer dimers as shown by single peak melting curves for individual genes (Fig. 2). The amplification efficiencies of all ten candidate reference genes were between 93% and 105%. The amplification efficiencies were 93% for *RPL4*, 94% for *SDHA*, 97% for each of *ACTB, HMBS* and *TBP*, 100% for *HPRT1*, 101% for *18 S rRNA*, 104% for *YWHAZ* and 105% for each of *ALB* and *GAPDH*. The correlation coefficient (R^2^) of all the standard curves performed in 6-point dilutions of RNA, ranged from 0.99253 to 0.99980. The overall expression patterns (Cq values) for these ten reference genes in the shell gland and spleen are shown in Fig. 3a,b, respectively. In the shell gland, most of the reference genes were highly expressed, with average Cq values between 15 and 21 cycles, except *18 S rRNA* and *ALB*, which showed average Cq values around 6 and 26 cycles, respectively (Fig. 3a). In the spleen, the average Cq values of the genes ranged from 12 to 21 cycles except for *18 S rRNA* that showed average Cq values around 4 (Fig. 3b). Both in the shell gland and in the spleen, the expression pattern of the ten reference genes was calculated in the combined data set of control, IBV T and Vic S strains challenged groups.

### Reference gene expression stability in shell gland and spleen

In the shell gland, in the combined data set of control, IBV T and Vic S strain challenged groups, the two most stable reference genes in geNorm and NormFinder were *TBP* and *YWHAZ* (Table 1). In the same data set, BestKeeper ranked *HMBS* and *RPL4* as the two most stable genes. The comprehensive ranking (geomean) showed *TBP* and *YWHAZ* as the two most stable genes. Despite slight variations in gene ranking in different software, the average expression stabilities (geNorm M) of the ten reference genes in the combined data set were within the acceptable range (<0.50) and varied from 0.159 (*YWHAZ*) to 0.373 (*ALB*) (Table 1). The two least stable genes in geNorm and NormFinder were *ACTB* and *ALB*, while BestKeeper showed *SDHA* as the least stable gene instead of *ACTB* (Table 1). The two least stable genes obtained in the comprehensive ranking were *SDHA* and *ALB*. The pairwise variation (geNorm V) recommended *TBP* and *YWHAZ* as the best set of genes with geNorm V (V2/3) value as 0.048 (Fig. 3c). A geNorm V value < 0.15 for the combination of first two genes showed that there was no need to combine the third gene to be used as set of reference genes for expression data analysis.

**Table 1:** Overall stability values of reference genes in the combined data set of shell gland and spleen affected by IBV T and Vic S strain challenge.

Group	Rank	geNorm		NormFinder		BestKeeper		Geomean	
		Gene	M value	Gene	SD	Gene	SD	Gene	Value
Shell gland	1	*YWHAZ*	0.159	*TBP*	0.071	*HMBS*	0.156	*TBP*	1.817
	2	*TBP*	0.161	*YWHAZ*	0.111	*RPL4*	0.176	*YWHAZ*	2.410
	3	*18 S*	0.162	*RPL4*	0.115	*TBP*	0.179	*RPL4*	3.107
	4	*HPRT1*	0.180	*HMBS*	0.118	*ACTB*	0.207	*HMBS*	3.175
	5	*RPL4*	0.216	18 S	0.133	*GAPDH*	0.211	*18 S*	4.932
	6	*GAPDH*	0.235	*GAPDH*	0.154	*HPRT1*	0.216	*HPRT1*	5.518
	7	*SDHA*	0.250	*HPRT1*	0.166	*YWHAZ*	0.224	*GAPDH*	5.646
	8	*HMBS*	0.262	*SDHA*	0.166	*18 S*	0.242	*ACTB*	6.868
	9	*ACTB*	0.274	*ACTB*	0.186	*SDHA*	0.246	*SDHA*	7.958
	10	*ALB*	0.373	*ALB*	0.787	*ALB*	0.537	*ALB*	10.000
Spleen	1	*TBP*	0.233	*HMBS*	0.088	*YWHAZ*	0.227	*TBP*	2.000
	2	*YWHAZ*	0.248	*ACTB*	0.088	*TBP*	0.256	*YWHAZ*	2.289
	3	*ACTB*	0.260	*GAPDH*	0.190	*ACTB*	0.279	*ACTB*	2.621
	4	*HMBS*	0.312	*TBP*	0.246	18 S	0.304	*HMBS*	2.714
	5	*GAPDH*	0.331	*HPRT1*	0.252	*HMBS*	0.406	*GAPDH*	4.481
	6	*HPRT1*	0.352	*YWHAZ*	0.364	*GAPDH*	0.417	*18 S*	5.809
	7	*18 S*	0.390	18 S	0.441	*HPRT1*	0.517	*HPRT1*	5.944
	8	*SDHA*	0.516	*SDHA*	0.457	*SDHA*	1.049	*SDHA*	8.000
	9	*RPL4*	0.673	*RPL4*	0.694	*ALB*	1.218	*RPL4*	9.322
	10	*ALB*	0.900	*ALB*	7.130	*RPL4*	1.442	*ALB*	9.655
All together (shell gland and spleen)	1	*TBP*	0.154	*ACTB*	0.450	*YWHAZ*	0.557	*YWHAZ*	1.817
	2	*YWHAZ*	0.159	*HPRT1*	0.944	*TBP*	0.623	*TBP*	2.289
	3	*GAPDH*	0.161	*YWHAZ*	1.017	*GAPDH*	0.630	*ACTB*	3.302
	4	*ACTB*	0.191	*SDHA*	1.027	*HPRT1*	0.720	*GAPDH*	3.557
	5	*HMBS*	0.203	*GAPDH*	1.076	18 S	0.746	*HPRT1*	3.826
	6	*18 S*	0.217	*TBP*	1.105	*HMBS*	0.757	*SDHA*	6.073
	7	*HPRT1*	0.231	*RPL4*	1.725	*SDHA*	0.856	*HMBS*	6.214
	8	*SDHA*	0.261	*HMBS*	2.300	*RPL4*	1.286	*18 S*	6.463
	9	*RPL4*	0.317	18 S	3.020	*ACTB*	1.483	*RPL4*	7.958
	10	*ALB*	0.439	*ALB*	9.984	*ALB*	1.742	*ALB*	10.000

The Cq values were analysed in geNorm, NormFinder and BestKeeper and a comprehensive ranking (geomean) was calculated by assigning an appropriate weightage to individual gene ranking obtained in individual software. A total of 10 samples for each of the groups (IBV T, Vic S and control) in each tissue (shell gland or spleen) were processed for qPCR assay.

In the spleen, with the combined data set of control, IBV T and Vic S strain challenge groups, the two most stable reference genes were *TBP* and *YWHAZ* with slight variations in the ranking obtained in NormFinder (Table 1). The two least stable reference genes across all the statistical software were *RPL4* and *ALB* (Table 1). The pairwise variation recommended *TBP* and *YWHAZ* as the two most stable reference genes with geNorm V value as 0.089 (Fig. 3c). Based on the data combined from the shell gland and spleen, *TBP* and *YWHAZ* were ranked as the two most stable reference genes in geNorm and BestKeeper. In the same data set, NormFinder ranked *ACTB* and *HPRT1* as the two most stable reference genes. The two least stable reference genes varied in different software (Table 1). The pairwise variation recommended *TBP* and *YWHAZ* as the two most stable reference genes with geNorm V value as 0.05 (Fig. 3c).

### Reference gene validation in shell gland and spleen

**Figure 4:**
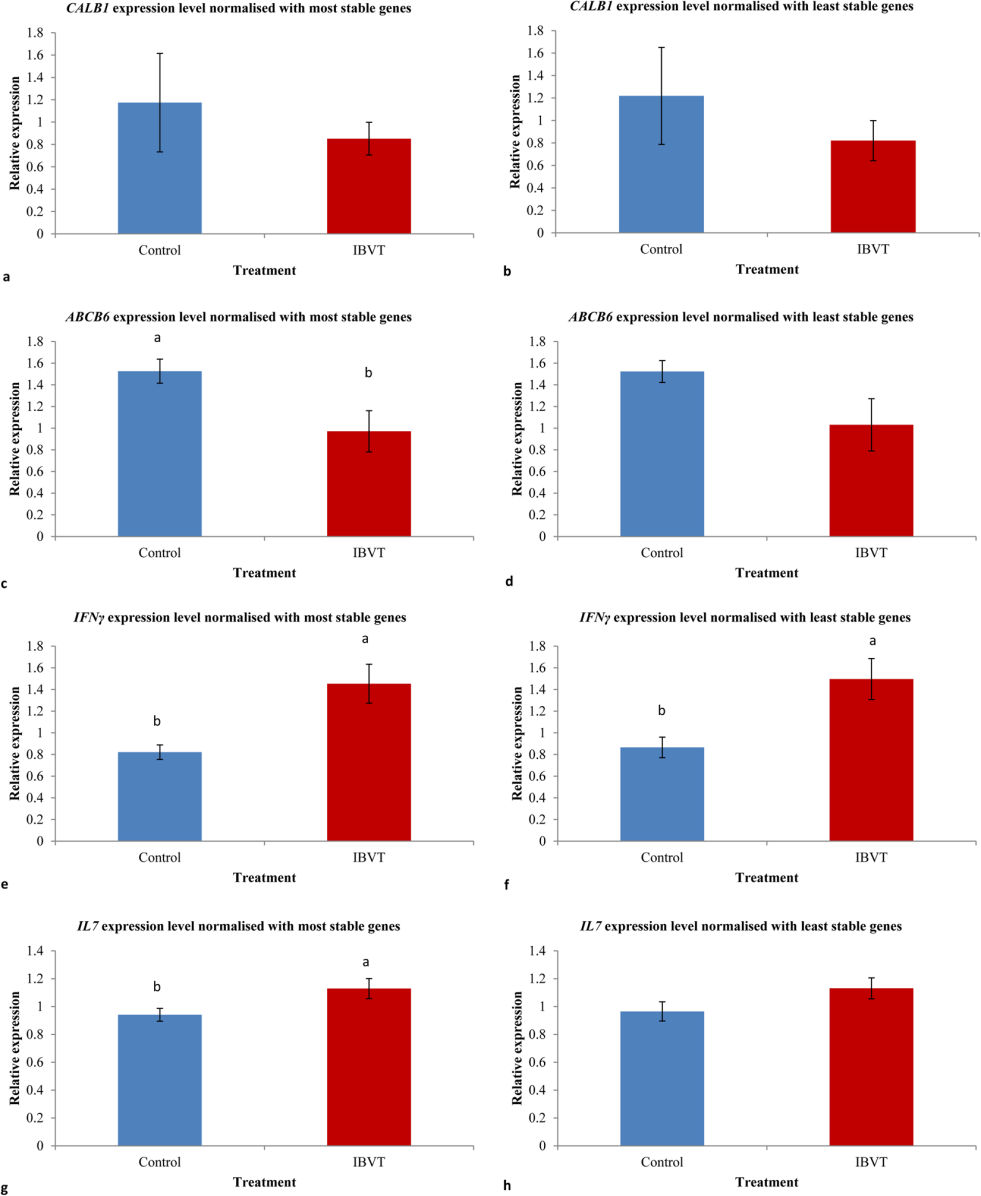
Relative expression levels of the candidate target genes affected by IBV T challenge in the shell gland and spleen of laying hens. (**a**) *CALB1* expression level in the shell gland normalized with the most stable genes *YWHAZ* and *TBP* (P = 0.6783). (**b**) *CALB1* expression level in the shell gland normalized with the least stable genes *SDHA* and *ALB* (P = 0.7788). (**c**) *ABCB6* expression level in the shell gland normalized with the most stable genes *YWHAZ* and *TBP* (P = 0.0152). (**d**) *ABCB6* expression level in the shell gland normalized with the least stable genes *SDHA* and *ALB* (P = 0.0713). (**e**) *IFNγ* expression level in the spleen normalized with the most stable genes *YWHAZ* and *TBP* (P = 0.0021). (**f**) *IFNγ* expression level in the spleen normalized with the least stable genes *RPL4* and *ALB* (P = 0.0050). (**g**) *IL7* expression level in the spleen normalized with the most stable genes *YWHAZ* and *TBP* (P = 0.0333). (**h**) *IL7* expression level in the spleen normalized with the least stable genes *RPL4* and *ALB* (P = 0.1130). Values are relative expression quantities and error bars show standard error. Across the treatment, ^a,b^show significant difference (p < 0.05). For the candidate target genes, normalised relative quantities (NRQ) were calculated in qbase + based on (${2}^{-{\rm{\Delta }}{\rm{\Delta }}\mathrm{Cq}}$) approach with a genes specific amplification efficiencies to show the relative expression of Cq levels in folds to the mean Cq of all samples of the genes. NRQ values were further analysed in Statview software (SAS) and Tukey-Kramer test was used to differentiate level of significance (p < 0.05) between means. A total of 20 samples for each of the groups (IBV T and control) in each tissue (shell gland or spleen) were processed for qPCR assay.

The relative expression levels of candidate target genes *CALB1* and *ABCB6* in the shell gland of laying hens challenged with IBV T strain were analysed with the two most stable (*TBP*, *YWHAZ*) and the two least stable reference genes (*SDHA*, *ALB*), according to the comprehensive ranking (geomean) applied in the study. The relative expression levels of *CALB1*, normalized with each of the two most stable and the least stable genes, were comparable with one another (Fig. 4a,b). However, the level of significance (p value) changed when the expression level of *CALB1* was normalised with the two most and two least stable reference genes. The expression levels of *ABCB6* were significantly affected when the comparison was made for the gene expression data normalized with the two most and least stable reference genes (Fig. 4c,d). The normalisation of the gene expression data with the two least stable reference genes led to erroneous interpretation of the data and the level of significance changed from P = 0.0152 to P = 0.0713. In the spleen, the relative expression levels of *IFNγ* and *IL7* were analysed with the two most stable (*TBP*, *YWHAZ*) and the two least stable reference genes (*RPL4*, *ALB*). The relative expression levels of *IFNγ* between the control and challenge groups, obtained from data normalisation with the two most stable reference genes, was comparable with the data normalised with the two least stable genes; however, the level of significance changed considerably (Fig. 4e,f). The relative expression levels of *IL7* normalised with the two most stable reference genes were significantly different between the IBV T and control groups, while the expression level of *IL7* became non-significant when the two least stable reference genes were used for normalisation (Fig. 4g,h).

## Discussion

We investigated the stability of ten reference genes in the shell gland region of the oviduct in laying hens when the egg was in the isthmus (5 hr post-oviposition time-point) region of oviduct in IBV T and Vic S strains challenged groups. The same set of genes was also investigated in spleen under the same IBV model. The current study provides information on the expression stability of these candidate reference genes and most stably expressed reference genes are suggested for the normalisation of gene expression data in the chicken shell gland and spleen in infectious bronchitis study models. In our previous study, *HPRT1* and *HMBS* were chosen as the two most stable reference genes in relation to three or four time-points of eggshell formation and nicarbazin treatment^45^. In the current study, the *HMBS* and *HPRT1* fell in the middle of the ranking order among the 10 reference genes ranked with the combination of data from both the shell gland and spleen. This study suggests that *YWHAZ* and *TBP* express more stably in the shell gland and spleen of laying hens compared with *HPRT1* and *HMBS*. As a general guideline, with geNorm ranking, it is stated that the benefit of using an extra (n + 1)th reference gene is limited as soon as the V_n/n+1_ value drops below the 0.15 threshold. In the current study, the geNorm V for the two most stably expressed reference genes was < 0.15 and adding the third most stable gene for expression data normalisation was not necessary. In gene expression studies performed on different tissues of chicken, *TBP*, *HPRT1* and *HMBS* have been reported to be among the most stable reference genes^46–48^. *RPL13* and *TBP* were ranked to be the most stable reference genes in chickens and turkeys infected with *Histomonas meleagridis* and fowl aviadenovirus^49^. However, in many other avian studies, differences in the stability of reference genes between the control and infected, among multiple avian species and between different tissues of the same species, were observed^46,49,50^. Similarly, in non-avian species, differences in the stability of reference genes raking in different tissues of the same species or organ developmental stages have been established^51–54^. Nevertheless, in the current study, the overall higher stability of *YWHAZ* and *TBP* across most of the software in the combination of different data sets indicated that these two genes can be used as reference genes for the normalisation of expression data in laying hens in IBV infection models. In the current study, in the combined data set of the shell gland samples, all ten reference genes were in the acceptable range as stable reference genes with geNorm M value < 0.5 and SD < 1.0. In the spleen samples, all genes but *SDHA*, *RPL4* and *ALB* showed SD < 1.0 in BestKeeper. This demonstrated that most of the genes analysed had relatively high stability both in the shell gland and spleen tissues in response to IBV challenge. A better stability value of *ACTB* in the spleen might indicate that this gene is more stable in spleen than in shell gland tissue. Recently, *ALB* and *ACTB* have been shown to be the least stable reference gene in various tissues of wild duck challenged with low pathogenic avian influenza A virus^55^. Similarly, *ACTB* has been reported as an unsuitable reference gene in *in vitro* studies of primary mouse fibroblasts treated with tumour extract^56^. In different vertebrate species, different reference genes under different treatments have been validated in the reproductive system. *GAPDH* and *HPRT1* were ranked as the two most stable reference genes in the ovary of geese^57^. In bovines, the two most stable reference genes in the uterus were *GAPDH* and *YWHAZ* ^58^. In the ovary of a mouse, under various toxicological treatments, the two most stable reference genes were *GAPDH* and *RPL13a* ^59^. *GAPDH* is involved in the glycolytic pathway and its expression depends on tissue type^60,61^ and certain conditions, such as deprivation of glucose and stress induction^62^. Recently, *GAPDH* was found to be the least stable reference gene in the inguinal white adipose tissue and skeletal muscle of caloric restricted mice^54^. In the current study, both in the shell gland and spleen, the *GAPDH* ranking was in the middle order across most of the statistical tools. Therefore, it is recommended not to use *GAPDH* as a reference gene for expression data normalisation until it has been tested under the prevailing experimental conditions, as MIQE guidelines recommend that the validation of reference genes should be performed before their use in expression data normalisation^63^.

Available literature on reference gene stability in human and various other animal species show variable results for *TBP* and *YWHAZ*. *TBP* was shown to be among the most stable reference genes in human glioblastoma samples^64–66^. In rat endogenous cardiac stem cell culture, *TBP* was among the least stable reference genes^67^. *YWHAZ* was among the least stable reference genes in various tissues of goat^68^. *TBP* was the least stable gene in different tissues of mouse^54^ and rat^59^. From the available literature, it seems that *TBP* and *YWHAZ* may not be the most stable reference genes in certain tissues of various species. Nevertheless, the current study confirms that *TBP* and *YWHAZ* are stably expressed genes in chicken tissues undergoing viral multiplication.

The present study has demonstrated that the rankings of the expression stability of 10 candidate reference genes had similar trends but discrepancies were observed among different statistical programmes used. Differences in gene ranking were also observed between shell gland and spleen. Similar discrepancies have been observed in our previous study^45^ and elsewhere with different species and treatments^48,57,69^. With different algorithms in different programmes, slight change of gene stability orders can be expected by the analyses using these programmes. Furthermore, the reference gene validation with the two most stable and two least stable reference genes showed that the least stable genes significantly affect the outcome of expression data normalisation and may lead to erroneous interpretation of such data.

In summary, we have performed optimisation of reference genes in samples collected from the shell gland and spleen tissues in IBV challenged laying hens. Two most stably expressed reference genes, *YWHAZ* and *TBP* have been chosen for the normalisation of gene expression data in the shell gland and spleen of chickens under IBV infection models in poultry and other avian species. The validation study has confirmed that the use of these two genes as reference genes led to discrimination between the expression levels of four candidate target genes upon different treatments, while the use of two least stable reference genes may lead to incorrect interpretation of data.

## Methods

### Animal Ethics

The experimental setup was approved by the University of New England, Animal Ethics Approval Committee under Authority No. AEC15-118. The protocol was carried out in accordance with the guidelines specified in Australian Code for the Care and Use of Animals for Scientific Purposes 8^th^ edition 2013.

### Rearing of IBV free laying hens and virus challenge

Day old Isa-Brown laying chickens were obtained from the Baiada Hatchery at Tamworth, NSW, Australia. At day-old, all the chickens received vaccine (Rispens) against Marek’s disease at the hatchery but were not vaccinated against infectious bronchitis (IB). The chickens were raised in isolation sheds at the University of New England under a strict biosecurity system. All chickens were fed commercial starter mash up to 4 weeks of age, pullet grower to 18 weeks of age and layer mash until the termination of the experiment. Pullets were moved from the isolation sheds at 18 weeks of age to individual cages in an isolated poultry house. There was no morbidity or mortality from the rearing period until challenge by IBV T and Vic S strains. Before challenge, an ELISA was performed to confirm that all birds were negative for IBV antibody titre in the blood. At 35 weeks of age, eggs were collected and processed for traditional egg quality measurements. Hens were divided into 2 × 3 factorial design (Table 2) in such a way that the egg weight and eggshell colour (L*) were not significantly different among the groups. The hens selected for IBV T and Vic S strain challenge were moved to a separate poultry house one week prior to challenge to settle down and recover from the trans-location stress. From the control hens, eggs were analysed until the hens were euthanised for shell gland and spleen tissues collection. At the time of euthanasia, the egg in individual hens was in the distal magnum/isthmus (5 hr post-oviposition time). At this oviposition time, the egg is getting ready to enter to shell gland tissue and thus it is assumed that the secretory activities in the shell gland will be commencing. From the challenged hens, eggs were analysed for 2 days prior to challenge and daily collection of eggs for analysis continued until individual hens were euthanised. In the challenged groups, 5 hens from each group at a time were intra-occularly inoculated with 10^7^ embryo infective dose (E.I.D_50_) and closely monitored for the development of clinical signs of IB and loss in eggshell colour until days 9–10 post-infection (p.i.). The E.I.D_50_ dose was determined in embryonated SPF eggs infected at 9 days of incubation with 10-times serial dilutions (10^−3^ to 10^−8^ dilutions) of IBV T strain. Eggs were opened at 16 days of incubation and the number of live, dead or virus affected (dwarfed, curled) embryos recorded. The 10^7^ E.I.D_50_ dose for IBV Vic S strain was calculated from the dose on the vial of commercial freeze-dried vaccine (Poulvac Bron Vic S, Zoetis Australia). The infection in challenged birds was confirmed through RT-qPCR (shell gland and spleen tissues) and ELISA (ELISA kit, IDEXX Laboratories, Inc., Westbrook, MA, USA).

**Table 2:** Grouping of laying hens for tissues collection for reference genes study.

Variable	No. of hens (n = 10)	Shell gland	Spleen
Control	+	+	+
IBV T challenge	+	+	+
IBV Vic S challenge	+	+	+

The control group was the same for both the IBV T and Vic S strains challenge groups.

### Selection of primers sequence and validation

In the current study, all ten reference genes were selected from the literature published for chickens (Table 3). Specific amplifications of the primers were assessed by generation of a single peak of melting curve using uMelt web based tool for predicting DNA melting curves and denaturation profiles of PCR products^70^. Furthermore, primer specificity was confirmed by obtaining a single band of appropriate size in Agilent 2100 Bioanalyzer (Agilent Technologies, Waldbronn, Germany) using Agilent DNA 1000 Kit per the manufacturer’s instructions. PCR amplification efficiencies and correlation coefficients (R^2^) were determined with the amplifications of a series of six 10-fold dilutions of RNA based on the following equation^71–74^;

$$E=({10}^{-(\frac{1}{-slope})}-1)\times 100$$

**Table 3:** Candidate reference and target genes in expression studies by qPCR in the shell gland and spleen of laying hens challenged with infectious bronchitis virus T and Vic S strains.

Gene name	Gene symbol	Primer sequence (5ʹ-3ʹ)	Amplicon size (bp)	Ta °C	PCR efficiency (%)	Correlation coefficient (R^2^)	Slope	Accession No.	Reference
Nuclear ribosomal RNA small subunit	*18 S rRNA*	F: TGTGCCGCTAGAGGTGAAATT	63	60	101	0.99873	−3.288	AF173612.1	Kuchipudi *et al*.^77^
		R: TGGCAAATGCTTTCGCTTT							
β-actin	*ACTB*	F: CTGTGCCCATCTATGAAGGCTA	139	60	97	0.99980	−3.387	NM_205518.1	Yang *et al*.^78^
		R: ATTTCTCTCTCGGCTGTGGTG							
Albumin	*ALB*	F: CCTGGACACCAAGGAAAT	197	60	105	0.99253	−3.009	NM_205261.2	Yang *et al*.^78^
		R: TGTGGACGCCGATAGAAT							
Glyceraldehyde 3-phosphate dehydrogenase	*GAPDH*	F: GAAGCTTACTGGAATGGCTTTCC	66	60	105	0.99874	−3.217	NM_204305.1	Kuchipudi *et al*.^79^
		R: CGGCAGGTCAGGTCAACAA							
Hydroxymethylbilane synthase	*HMBS*	F: GGCTGGGAGAATCGCATAGG	131	60	97	0.99953	−3.397	XM_417846.2	Yin *et al*.^80^
		R: TCCTGCAGGGCAGATACCAT							
Hypoxanthine Phosphoribosyltransferase	*HPRT1*	F: ACTGGCTGCTTCTTGTG	245	63	100	0.99870	−3.322	NM_204848.1	Yang *et al*.^78^
		R: GGTTGGGTTGTGCTGTT							
Ribosomal protein L4	*RPL4*	F: TTATGCCATCTGTTCTGCC	235	60	93	0.99785	−3.502	NM_001007479.1	Yang *et al*.^78^
		R: GCGATTCCTCATCTTACCCT							
Succinate dehydrogenase complex flavoprotein subunit A	*SDHA*	F: TCTGTCCATGGTGCTAATCG	126	60	94	0.99790	−3.484	NM_001277398.1	Bages *et al*.^46^
		R: TGGTTTAATGGAGGGGACTG							
TATA-Box Binding Protein	*TBP*	F: TAGCCCGATGATGCCGTAT	147	61	97	0.99676	−3.407	NM_205103	Li *et al*.^81^
		R: GTTCCCTGTGTCGCTTGC							
Tyrosine 3-monooxygenase/tryptophan 5-monooxygenase activation protein, zeta polypeptide	*YWHAZ*	F- TTGCTGCTGGAGATGACAAG	61	60	104	0.99912	−3.222	NM_001031343.1	Bages *et al*.^46^
		R- CTTCTTGATACGCCTGTTG							
Calbindin 1	C*ALB1*	F: TTGGCACTGAAATCCCACTGA	116	60	100	0.99873	−3.322	NM_205513.1	Qi *et al*.^82^
		R: CATGCCAAGACCAAGGCTGA							
ATP binding cassette subfamily C member 6	*ABCB6*	F: CTCAACTGGTTCGGCACCTA	107	60	105	0.99761	−3.150	XM_015290086.1	this study
		R: TTCACTGCATCCTTCACCTCC							
Interferon gamma	*IFNγ*	F- GTGAAGAAGGTGAAAGATATCATGGA	71	60	99	0.99878	−3.180	NM_205149.1	this study
		R- GCTTTGCGCTGGATTCTCA							
Interleukin 7	*IL7*	F- GGTTCTGCCACTTCTCCTTG	160	60	103	0.99554	−3.255	NM_001037833.1	this study
		R- CTTGCAGCATCTGTCACGATA							

For calculating amplification efficiency, a standard curve was generated using a 10-fold dilution of RNA amplified on the Rotor-Gene Q thermocycler real-time system. Standard curve was obtained by plotting the Cq values against the log of the starting quantity of template for each dilution.

The qPCR was performed on the reference genes when the PCR amplification efficiency was in a range of 93 to 105%, and linear correlation coefficient R^2^ > 0.980 were considered of high standard^63,73^.

### Tissue collection for RNA extraction

Hens were humanely euthanised with CO_2_ gas and the shell gland was aseptically retracted through the abdominal incision. The shell gland was opened from the anterior-ventral side and an approximately 500 mg sample tissue was cut from the centre of the shell gland and directly transferred to RNALater (Sigma Aldrich, Australia) in 2 mL Eppendorf tubes. The samples were stored at −20 °C and were processed for total RNA extraction within one day of collection. At the same time of shell gland tissue collection, an approximately 500 mg sample of spleen was cut in such a way that it contained both red and white pulp and was directly transferred into RNALater and processed as described earlier.

### Total RNA extraction and purification

Total RNA was extracted using TRIsure (Bioline, Australia), according to the manufacturer’s instructions. Briefly, an approximately 100 mg of tissue (wet weight) was homogenized in 1 mL of TRIsure using an IKA T10 basic Homogenizer (Wilmington, NC, USA). After the RNA pellets were washed with 1 mL ethanol (75%), 50 µL of UltraPure™ DEPC-treated water (Ambion, USA) was used to dissolve RNA pellets. The total RNA was further purified using RNeasy Mini Kit (Qiagen, GmbH, Hilden, Germany) as per the manufacturer’s instructions. A DNase-I step was included to get rid of the genomic DNA. The elution of RNA from the spin column with 50 μL of RNase-free water was repeated twice and the eluted RNA solutions were mixed thoroughly. The purified RNA was analysed in a NANODROP-8000 spectrophotometer (ThermoFisher Scientific, Wilmington, DE, USA) to measure its quantity and purity. RNA integrity and purity were also examined in Agilent 2100 Bioanalyzer using Agilent RNA 6000 Nano Kit as per the manufacturer’s instructions. All the RNA showed distinct 18 S and 28 S bands with an average RNA integrity number (RIN) > 9.1.

### Quantitative PCR

qPCR was performed with the SensiFAST SYBR^®^ Lo-ROX One-Step RT-PCR Kit (Bioline, Australia). Master mix was prepared as per the manufacturer’s protocol and RNA template from 1:100 dilutions was added to the reaction wells using QIAgility robotic (Qiagen, Australia). The reaction in a volume of 20 µL contained 10 µL of 2 × SensiFAST SYBR low Rox one-step mix, 400 nMoles primers, 0.2 µL of reverse transcriptase, 0.4 µL of RiboSafe RNase inhibitor, 3.8 µL RNase-free water and 4 µL of RNA template. The reaction was run in triplicates in a Rotor-Gene Disc 100 (Qiagen, Australia) with a Rotor-Gene Q thermocycler (Qiagen, Australia). No template control (NTC) and no reverse transcriptase (-RT) control were also included to detect possible contamination. Thermocycling conditions for a 2-step PCR were: reverse transcription at 45 °C for 10 minutes, first denaturation at 95 °C for 2 minutes, then 40 cycles of denaturation at 95 °C for 5 s and annealing at 60 °C, 61 °C or 63 °C for 20 s.The fluorescent data were acquired at the end of each annealing step during PCR cycles. A melting step was conducted to assess the specificity of PCR amplification. The qPCR products were examined in the Bioanalyzer using Agilent DNA 1000 Kit to determine the amplification specificity by the size of the amplicons estimated.

### Statistical Analysis

The geNorm module in qbase + software (version 3.0) was used to calculate the gene expression stability measure (geNorm M)^34,39^. The input data for qbase + were generated using the relative quantities based on comparative quantification cycle (Cq). In addition, the raw Cq values were analysed in NormFinder (GenEx version 6.0.1)^40,41^ for reference gene expression stabilities. NormFinder calculates the standard deviation (SD) of the genes relative to the mean expression of all the genes in the panel. Before data analysis in qbase + and NormFinder, the data are pre-processed for quality control, such as inter-run calibration, amplification efficiency correction, missing data handling, failing replicates (>0.5 Cq difference) removal and conversion to mean relative quantities. An Excel based BestKeeper (Version 1.0) software was used to determine the most stable reference genes based on Pearson correlation coefficient (r), coefficient of variance (CV) and standard deviation^42^. The overall ranking of the 10 reference genes was calculated by assigning an appropriate weightage value to individual gene ranking obtained in the three different statistical applets^75^. The principles of individual software (algorithms) have been detailed in the introduction section.

For reference gene validation, relative expression levels of candidate target genes *CALB1*, *ABCB6*, *IFNγ* and *IL7* genes were calculated by the comparative 2^−ΔΔCq^ approach^71,76^ in qbase + software (version 3.0)^39^, using the two most stable (*YWHAZ* and *TBP*) and the two least stable reference genes (*ALB* and *SDHA*/*RPL4*). From the qbase + , normalized relative quantities (NRQ) values were further analysed with One-way ANOVA in Statview Version 5.0.1.0 (SAS Institute Inc., 1998) and Tukey-Kramer test was used to differentiate level of significance (p < 0.05) between means.
